# Urban Phytoremediation: A Nature-Based Solution for Environmental Reclamation and Sustainability

**DOI:** 10.3390/plants14132057

**Published:** 2025-07-04

**Authors:** Luca Di Stasio, Annamaria Gentile, Dario Nicola Tangredi, Paolo Piccolo, Gianmaria Oliva, Giovanni Vigliotta, Angela Cicatelli, Francesco Guarino, Werther Guidi Nissim, Massimo Labra, Stefano Castiglione

**Affiliations:** 1 Department of Chemistry and Biology “A. Zambelli”, University of Salerno, 84084 Fisciano, SA, Italy; 2 NBFC, National Biodiversity Future Center, 90133 Palermo, PA, Italy; 3 Department of Biotechnology and Biosciences, University of Milano Bicocca, 20126 Milan, MI, Italy

**Keywords:** urban brownfields, emerging pollutants, organics and inorganics, urban sustainability, phytotechnologies

## Abstract

Starting from the Industrial Revolution in the 18th century to date, urban areas have faced increasing environmental challenges due to the diffusion of harmful substances, resulting from vehicular traffic, the activities of different industries, waste, and building construction, etc. These pollutants are dangerous, since they pose a threat to both the environment and human health. Phytoremediation is an environmentally friendly and low-cost technique that uses plants and their associated microorganisms to clean-up contaminated sites. In this review, we explore its main applications in urban settings. Specifically, we investigate how phytoremediation works, highlighting the most effective plants for its success in a city context. Moreover, we also describe the main factors influencing its effectiveness, such as soil, climate, and pollutants. In this regard, several case studies, conducted worldwide, have reported on how phytoremediation can successfully reclaim contaminated areas, transforming them into reusable city green spaces, with reduced costs compared to traditional remediation techniques (e.g., soil replacement, soil washing, etc.). Moreover, by integrating it into urban planning, cities can address environmental pollution, while promoting biodiversity, enhancing the landscape, and increasing its social acceptance. This nature-based solution offers a practical path toward more sustainable and resilient urban environments, especially in regard to the climate change framework.

## 1. Introduction

Urban environments are dynamic spaces, wherein the interplay of human activities, infrastructure, and natural elements shapes modern cities. These areas are hubs of economic development, cultural exchange, and innovation, accommodating diverse populations and fostering social interactions. However, urban settings also face challenges, such as overcrowding, environmental degradation, and the need for sustainable resource management. Since cities continue to expand and evolve, understanding their complexities is essential for the creation of pleasant and livable places.

The rapid expansion of urban areas has led to the intensification of various anthropogenic activities [1], contributing to the development of 3.5 billion potentially polluted sites in Europe alone [2]. In such a context, the contamination of urban areas, especially soil, is a serious environmental issue that poses significant risks to human health, ecosystems, and the sustainability of cities.

Urban soils can be found in the proximity of buildings, industrial zones, city parks, waste disposal sites, and peri-urban agricultural lands. For these reasons, they are frequently contaminated with numerous toxic substances, such as organic and/or inorganic pollutants, pesticides and, in some cases, even radioactive materials.

In urban environments, there are several sources of soil contamination, as follows:

- Industrial activities: Factories and manufacturing plants often release hazardous substances, including heavy metals (lead, cadmium, mercury, etc.), organic pollutants, such as polychlorinated biphenyls (PCBs) or polycyclic aromatic hydrocarbons (PAHs), and other toxic chemicals [3]. All these substances can leach into the soil, contaminating it and potentially entering aquifers first, and then entering into the food chain.
- Vehicular traffic: Motorcycles, cars, busses, and articulated trucks contribute to soil contamination through the deposition of exhaust emissions, tire and brake wear particles, and road runoff [4]. These pollutants can accumulate on roads, along roadsides, and in nearby soils, seriously affecting their quality and biological diversity.
- Inadequate waste disposal: The improper disposal of municipal, industrial, and hazardous wastes is another significant source of soil contamination [5]. Landfills and dumpsites, if not handled properly, can leak toxic leachates into the surrounding soil and groundwater. Additionally, the illegal dumping of waste in vacant lots and along waterways can lead to localized soil contamination.
- Agricultural practices: The use of pesticides, herbicides, and fertilizers in urban agriculture and gardening can contribute to soil contamination [6]. All these chemicals can persist for a long time in the soil, affecting its characteristics and, potentially, even contaminating crops.
- Construction activities: Construction and demolition activities can disturb and expose contaminated soil layers, releasing pollutants into the environment. Moreover, their use in the construction of buildings, houses, and roads can lead to the spread of pollutants across urban landscapes [7].
- Soil contamination in urban areas can have far-reaching consequences. It can impair soil functions, reduce agricultural productivity, contaminate groundwater resources, and negatively affect human health through direct exposure or the consumption of either contaminated food or water. Additionally, polluted soils can harm wildlife and disrupt ecosystem services, further degrading city environments.

Urban soil contamination poses significant challenges for many cities, particularly as they try to strike a balance between development and environmental restoration. Cities worldwide are facing the challenge associated with the need to restore degraded areas, commonly referred to as brownfields, which include former industrial sites, abandoned spaces, or decommissioned landfills. These sites often carry the legacy of years of pollution, due to the dispersal of HMs, hydrocarbons, and many other industrial byproducts, which are embedded in the soil. Such contamination not only poses environmental and health risks, but also limits their usability, which means that they are often left as blighted patches of land within urban landscapes.

Urban planners and environmental scientists are focusing on these neglected areas, with the aim of transforming them into safe, productive, and reusable green spaces. Beyond addressing the environmental hazards, reclaiming these brownfield sites also enhances urban livability and aesthetics, turning potential liabilities into assets. The ecological transition process requires innovative solutions, and successful interventions can revitalize entire neighborhoods, attracting investment, boosting urban biodiversity, and providing new green spaces for public enjoyment [8].

One promising approach in this context is phytoremediation, which utilizes plants, often in association with rhizospheric and endophytic microorganisms, to extract, contain, or breakdown contaminants present in the soil, offering a sustainable and visually pleasing approach to urban soil recovery. Phytoremediation is a green, cost-effective, simple, and aesthetically pleasing strategy [9], and it is also considered to be an eco-friendly alternative to traditional chemical–physical and engineering remediation methods [10]. In particular, it has the peculiarity of preserving the biological and pedological integrity of reclaimed soil [11]; in fact, phytoremediation improves the biological fertility of the soil and even reduces its erosion, thanks to well-developed root systems in the soil [12]; limits soil temperatures [13]; positively affects biogeochemical cycles of elements; improves urban biodiversity [14]; and, last but not least, it benefits urban CO_2_ sequestration. Moreover, city green spaces also have the characteristic of reducing vehicular traffic noise [15] and add a positive aesthetic value, which frequently increases the overall acceptability of the intervention, thanks to broader public support [16]. In addition, phytoremediation may provide important ecosystem services, providing biomass that can, eventually, be converted into bioenergy [8]. Given the multifaceted nature of soil contamination in urban areas and its detrimental impacts, there is an urgent need for effective remediation strategies. Therefore, phytoremediation, with its potential to address these challenges in a sustainable and cost-effective manner, offers a promising solution.

The main purpose of the current review is to analyze the state of the art and very successful case studies involving phytoremediation, employed for cleaning up and redeveloping contaminated urban sites. By synthesizing up-to-date research, this review aims to provide a comprehensive overview of the mechanisms, methodologies, and applications of phytoremediation in urban settings. Moreover, it aims to identify the challenges and limitations of this technology and highlight potential areas for further research and development. In the following sections, we will explore the various phytoremediation strategies, the choice of plant species, the optimization of the growth conditions, and its efficiency and effectiveness. Furthermore, this review also estimates the economic and environmental benefits of phytoremediation, including its potential to revitalize brownfields and contribute to sustainable urban development. Moreover, the case studies about urban phytoremediation discussed in this review serve as tools to fill the gap between theory and practice, with the aim of guiding the replication and scaling up of successful interventions. By identifying gaps in the current literature and outlining future research perspectives, this review also aims to guide scientists and policy makers in advancing the field of phytoremediation and promoting its wider adoption for the redevelopment of abandoned or contaminated urban sites.

## 2. Urban Soil Pollutants

Urban soils represent a complex and dynamic environmental compartment that is often subjected to significant contamination due to the intensity and diversity of human activities. The most common contaminants in urban soils and their concentrations are presented in Table 1, and they include HMs, organic pollutants, emerging contaminants, and excess nutrients, all of which result from a combination of industrial processes, transportation systems, construction activities, waste mismanagement, and inappropriate agricultural practices [17].

### 2.1. Heavy Metals and Trace Elements

Heavy metals (HMs) and trace elements (TEs), such as lead (Pb), cadmium (Cd), arsenic (As), mercury (Hg), copper (Cu), and zinc (Zn), are among the most pervasive inorganic pollutants in urban soils [18]. Their sources include vehicular emissions (e.g., brake pads, tires, and exhausts), industrial discharges, the use of lead-based paints, and atmospheric deposition from coal combustion. These elements are characterized by their persistence in the environment, since they do not degrade over time, and also by their high toxicity, which can severely affect soil qualities [19]. Lead, for instance, is a particular issue in old urban areas with a history of industrial activity or the widespread use of leaded gasoline and paints. Cadmium and arsenic, on the other hand, often accumulate due to the improper disposal of industrial and electronic waste.

HMs can bind strongly to soil particles, especially in clay or organic matter, but, under certain conditions (such as acidity), they may leach into the groundwater or become bioavailable to plants and microorganisms, consequently entering into the food chain [20]. This creates a potential pathway for exposure to humans and animals, amplifying the risks associated with contaminated urban soils.

### 2.2. Organic Pollutants

Urban soils are severely contaminated by organics, including PAHs, PCBs, dioxins, and hydrocarbons from petroleum sources. These compounds are introduced into the environment through the incomplete combustion of fossil fuels, spills of petroleum and chemicals, urban runoff, and the illegal dumping of industrial waste. PAHs, for instance, are byproducts of combustion from vehicles, industrial plants, and heating systems, and they are known to the health authorities for being carcinogenic and mutagenic [21]. Similarly, PCBs and dioxins, despite them being banned in many countries, are still present and persist in the environment, due to their resistance to degradation and legacy pollution from historical usage.

Urban soil contamination with organic pollutants often occurs in hotspots on the sides of roads, inside industrial sites, and near landfills. These pollutants are lipophilic, meaning they tend to accumulate in soil organic matter, making their remediation challenging. They also pose risks to soil microorganisms, potentially disrupting the soil’s ability to support ecosystem services, such as nutrient cycling and water reclamation and filtration [22].

#### Emerging Contaminants

In recent years, attention has turned to emerging contaminants in urban soils, such as microplastics, pharmaceutical residues, personal care products, and nanomaterials. Microplastics, derived from the fragmentation of larger plastic debris or synthetic fibers, are increasingly detected in urban soils, particularly in recreational parks, green spaces, and areas receiving stormwater runoff. These particles can alter the soil structure, reduce water infiltration, and affect microbial communities [23]. Pharmaceutical residues and personal care products often enter soils through the application of sewage sludge as fertilizer, through leaching from landfills, or wastewater effluents. While their long-term effects on soil ecosystems are still being studied, many of these compounds have been found to exhibit endocrine-disrupting properties and can adversely impact soil-dwelling organisms. Exposure to antibiotics, for instance, can lead to the antibiotic resistance phenomenon, which occurs when bacteria or other microorganisms develop the ability to resist the antibiotic, making these drugs less effective or even ineffective in treating infection diseases, which is emerging as a growing threat to human and animal health and is all the more reason to deal with its existence in the environment [24].

### 2.3. Excess of Nutrients and Agrochemicals

Urban soils frequently receive excess inputs of nutrients and agrochemicals, such as nitrogen and phosphorus from fertilizers, as well as pesticides, employed in urban gardening and landscaping [6]. Although these inputs are often intended to enhance crop productivity and vegetation growth, their overuse can lead to nutrient imbalances, reduced soil biodiversity, and the contamination of nearby water bodies through runoff and leaching. Pesticides and herbicides may also persist in soils for a long time, potentially harming non-target organisms, such as earthworms and many other beneficial terrestrial animals and microorganisms.

**Table 1:** Contaminants commonly found in urban soils, sources, and their known concentrations.

Pollutants	Examples	Sources	Concentrations	References
HMs	Lead (Pb)	Lead-based paints, vehicular emissions, activities of different industries	29–25,380 mg kg^−1^	[18]
	Cadmium (Cd)	Batteries, fertilizers, industrial waste	0.15–9 mg kg^−1^	
	Mercury (Hg)	Industrial processes, electronic waste	0.1–1 mg kg^−1^	
	Chromium (Cr)	Industrial waste, leather tanning processes	23–195 mg kg^−1^	
	Arsenic (As)	Pesticides, chemical products, mining activities	6–15 mg kg^−1^	
Hydrocarbons	PAHs (polycyclic aromatic hydrocarbons)	Fossil fuel combustion, vehicular traffic	90–52,000 μg kg^−1^	[21]
Pesticides and Agrochemicals	Herbicides, insecticides, fungicides, disinfectants	Agricultural use, pest control, urban garden maintenance	0.1–10 mg kg^−1^	[6]
Emerging Contaminants	Pharmaceuticals (e.g., antibiotics), personal care products	Disposal of medical waste, wastewater effluent, improper waste disposal, cosmetics, urban runoff	100 ng kg^−1^ to 2600 μg kg^−1^	[24]
	Microplastics	Plastic waste, tire wear	1000–10,000 particles kg^−1^	[23]

Urbanization also leads to physical and chemical alterations of soil properties that exacerbate contamination issues. Soil compaction, caused by construction activities, reduces soil permeability, due to the sealing of surfaces with asphalt and concrete. Moreover, the changes in soil pH from industrial emissions influence the mobility, bioavailability, and distribution of contaminants. In some cases, contaminants may even migrate altogether from soil to air or water systems, contributing to the broadening and increasing of environmental pollution [22].

Direct human exposure can occur through inhalation of contaminated dust, the ingestion of soil particles, or the consumption of crops grown on contaminated soils. Children are at higher risk due to their frequent contact with soil during play activities [19].

Given the heterogeneity of urban soils and the interplay of multiple contamination sources, effective management and remediation require integrated approaches. These include regular monitoring to assess contaminant levels, the application of phytoremediation or bioremediation techniques, and the implementation of stricter regulations to control pollutant sources.

## 3. Phytoremediation

### 3.1. Phytoremediation Technologies

Phytoremediation is a sustainable and eco-friendly remediation strategy that harnesses the synergistic interactions between plants and their associated microbial communities to restore contaminated environmental matrices. This approach capitalizes on the natural biochemical and physiological processes occurring in the rhizosphere to remove, transform, or immobilize a wide range of organic (e.g., PAHs, PCBs, phenols, and pesticides) and inorganic (e.g., heavy metals, metalloids, radionuclides, phosphates, and nitrates) pollutants across various matrices, including water, soil, sediments, and air [25].

The main phytoremediation mechanisms include phytoextraction, phytodegradation, rhizofiltration, phytostabilization, and phytovolatilization. These processes encompass the absorption and accumulation of contaminants in plant organs and tissues, their metabolic degradation, the filtration of pollutants through plant roots, the stabilization of contaminants in the rhizosphere, and the release of volatile substances into the atmosphere through transpiration.

Phytoextraction is an in situ remediation process that employs different plant species to absorb contaminants (specifically HMs and radionuclides) from the soil through their root systems, subsequently translocating and accumulating them in their above-ground organs (stems and leaves) [26].

Phytodegradation is a process that relies on plants and their associated microbial communities to absorb, metabolize, and degrade organic pollutants [27]. This mechanism involves the activity of plant-derived enzymes, such as dehalogenases and nitroreductases, that initiate the breakdown of complex organic compounds into simpler less toxic forms [28]. Plant-associated microorganisms, including rhizospheric and endophytic bacteria, play a complementary role by enhancing the biodegradation of contaminants in polluted soils. These bacteria benefit from root exudates (mucigel), which are rich in carbon sources, that stimulate their growth and metabolic activity, thereby accelerating contaminant degradation. In turn, they contribute to plant health by producing degradative and plant growth-promoting enzymes, which help mitigate pollutant-induced stress and support overall plant growth and fitness [29].

Rhizofiltration refers to the use of aquatic plants, whether free floating, submerged, or emergent, to remove organic and inorganic contaminants from water through absorption, adsorption, or precipitation, by means of their root systems. This technique is primarily applied to treat groundwater, surface water, and wastewater, with relatively low levels of pollution [30].

Phytostabilization is an in situ mechanism of remediation, whereby contaminants are inactivated or immobilized in the plant roots or the surrounding rhizosphere. The stabilizing action of plant roots restricts the mobility and availability of the contaminants, thereby reducing their toxic effect on the environment [31]. This mechanism does not generate contaminated secondary waste that needs further treatment, the contaminants are not removed from the site, but they remain there in a much less harmful form.

Phytovolatilization is a contaminant removal process that occurs through plant transpiration. In regard to this technique, pollutants from soil and water are absorbed by plants, or in cooperation with rhizosphere microorganisms, and then they are released into the atmosphere through leaf transpiration [32]. This strategy promotes the removal of pollutants, avoiding repeated plant pruning and the consequent disposal of the biomass produced.

The successful application of phytoremediation as an environmental remediation strategy requires a comprehensive understanding of the diverse mechanisms involved and their interplay with site-specific factors. The selection and integration of appropriate techniques depend on the nature of the contaminants, the environmental conditions, and the ecological characteristics of the impacted site. Additionally, considerations related to economic viability, operational feasibility, and long-term sustainability are paramount to ensure effective and resilient remediation outcomes are achieved [33].

### 3.2. Pollutant Removal Mechanisms

A thorough understanding of the physiological and biochemical mechanisms underpinning pollutant uptake, translocation, and detoxification in plants is fundamental to optimizing the phytoremediation strategies described above [34]. In such a context, plants are able to secrete into their rhizosphere chelators compounds such as phenolic and organic acids [35].

Chelating agents act by forming stable metal–chelate complexes, thereby enhancing the bioavailability of HMs in the soil and facilitating their uptake and translocation from the roots to the aerial parts of plants. This complexation increases metal mobility in the rhizosphere and improves their systemic transport within the plant. Based on previous studies, the application of chelating agents in phytoremediation technologies has been shown to significantly enhance the accumulation of HMs in various plant species, thereby improving the overall effectiveness of the remediation process [36].

Deng et al. [37] demonstrated that industrial hemp (*Cannabis sativa* L.) exhibited robust growth in Pb-contaminated mine soil when supplemented with various chelating agents (ethylenediaminedisuccinic acid—EDDS, ethylenediaminetetraacetic acid—EDTA, citric acid—CA, and nitrilotriacetic acid—NTA), which significantly enhanced Pb accumulation in plant organs.

Moreover, Yang et al. [38] showed that Cd extraction in *Zea mays* L. was improved by applying gibberellic acid and diethoxy succinic acid.

On the other hand, Peng et al. [39] reported that *Vallisneria natans* (Lour.) H. Hara and *Pistia stratiotes* L., when exposed to Cu^2+^ and Mn^2+^, produced significant amounts of caffeic acid, a phenolic chelating agent known to enhance metal mobility in soil and promote their uptake.

In addition to chelation, another strategy that plants adopt in terms of the phytoremediation process is exudation. Root exudation refers to the process by which plants secrete a diverse array of organic compounds from their roots into the rhizosphere. Root exudates are complex mixtures of low- and high-molecular-weight compounds that play key roles in the rhizosphere. They modify soil chemistry (pH, redox potential, nutrient availability) and stimulate microbial communities by providing either carbon or energy, indirectly enhancing the biodegradation of organic pollutants. Vacuole sequestration is a key plant defense that isolates HMs by storing them in the vacuole. This reduces their toxicity by keeping HMs away from the cytosol and organelles. For instance, plants like barley (*Hordeum vulgare* L.) and red fescue (*Festuca rubra* L.) store Zn in their vacuoles, in this way, aiding the cellular detoxification process [40].

Organic pollutants, such as PAHs, are easily absorbed by plants, but their removal is a concentration-dependent process [41,42]. However, the cellular and molecular mechanisms involved in their degradation by plants are not yet fully understood. It is known that the two main pathways of absorption are either airborne, via (air-to-plant) processes, or through the soil, via soil-to-plant processes. However, there are conflicting studies, as described by Chen et al. [43], on what the real main route of uptake of organic pollutants by plants may be.

Wang et al. [44] conducted a study on rice plants to assess the primary uptake pathways (root absorption, gaseous leaf uptake, or particle-bound deposition) for organophosphate esters, phthalates, and PAHs. This study revealed no single dominant pathway for all of the compounds studied. However, compounds with lower octanol–water partition coefficients (K_OW_) exhibited greater translocation from the roots to the aerial parts of plants, while those with higher octanol–air partition coefficients (K_oa_) were predominantly absorbed via particle deposition on the leaf surface.

In addition, a notable study was carried out by Alves et al. [45], who employed fluorescence microscopy to investigate the shoots and roots of *Medicago sativa* L. cultivated in PAH-contaminated soils (pyrene, anthracene, and phenanthrene). Their findings highlighted the potential role of glandular trichomes in the accumulation and possible degradation of contaminants.

### 3.3. Factors That Influence Phytoremediation Effectiveness

Environmental conditions play a critical role in determining the success of phytoremediation by directly influencing plant biomass, metabolic activity, and pollutant uptake. Factors, such as the climate, soil type, pH, and moisture, not only affect plant growth, but also the mobility and bioavailability of contaminants [46]. For instance, in cold regions, low temperatures can limit biomass production, while in arid environments, water scarcity can severely hinder remediation efforts. Consequently, site-specific environmental constraints must be considered when selecting suitable plant species, as these can significantly reduce the overall effectiveness of phytoremediation.

Temperature is a determinant for phytoextraction. Walne & Reddy [47] emphasized its influence on both biomass accumulation and HM uptake. Plant biomass generally increases with temperature, up to an optimum threshold; beyond this threshold, it may decline. Additionally, temperature, along with sunlight, rainfall, and wind, regulates transpiration rates, which, in turn, affects contaminant uptake [48]. Kudo et al. and Rahman et al. [49,50] reported that Cd uptake by *Peltigera horizontalis* (Huds.) Baumg. (1790) and *Dumortiera hirsuta* (Sw.) Nees (1833), increased with temperature, whereas As accumulation in *Pteris cretica* L. was not significantly influenced by thermal variations.

Phytoremediation performance depends not only on the climate, but also on soil pH and salinity. Soil pH governs the solubility, mobility, and uptake of nutrients and metals. Jerwin et al. [51] showed that reducing the soil pH from 7.0 to 5.0 more than doubled the Cu uptake in *Dendrocalamus asper* (Schult.) Backer. Similarly, Willscher et al. [52] demonstrated that the uptake of various HMs (Cd, Cu, Fe, Mn, Ni, Zn, Pb) by *Helianthus tuberosus* L. was significantly influenced by pH.

Moreover, high salt concentrations, common in coastal or degraded soils, lead to the accumulation of toxic ions, such as Na^+^ and Cl^−^, which disrupt plant growth and cellular functions [53]. According to Liu et al. [54], elevated salinity induces oxidative stress by promoting the formation of reactive oxygen species (ROS), potentially causing DNA damage and genomic instability. However, Zhu et al. [55] found that salinity could enhance HM uptake by increasing ion mobility, illustrating its dual role.

Analogous effects are observed in the case of organic pollutants. Soil pH alters the solubility and bioavailability of organic compounds, thus influencing both plant physiology and rhizospheric microbial activity [56]. In acidic soils, these contaminants tend to bind tightly to minerals and organic matter, reducing their bioavailability. In contrast, slightly alkaline conditions (pH 6.5–6.8) enhance microbial degradation and root uptake, supported by increased exudate production [57]. Moreover, rising temperatures can improve the solubility and mobility of organic pollutants, further facilitating their uptake and degradation by plants and associated microorganisms [58].

## 4. The Main Phytoremediation Actors

### 4.1. Plants

Based on the different phytoremediation strategies, the plant species are generally selected for their particular capabilities, such as rapid growth, large root system, high biomass production, and tolerance/resistance to the pollutants occurring at the site intended for reclamation. Certainly, among the most commonly used and recommended plant species for phytoremediation, the best performing ones for urban sites are: poplar, willow, sunflower, and some others [59].

Table 2 presents the most common plant species employed for phytoremediation purposes, as well as their main features in the presence of different pollutants.

#### 4.1.1. Phytoextraction Potential Indices

The bioconcentration factor (BCF) and the translocation factor (TF) are two indices that provide information about the phytoextractive potential of a plant related to ions. The BCF is expressed as the ratio between the concentration of an ion (e.g., pollutant) in the plant and its concentration in the soil [60]. The TF estimates the ion translocation efficiency from the roots to the aerial plant organs and it is calculated as the ratio between the concentration of an ion (e.g., pollutant) in the shoots and its concentration in the roots [61].

#### 4.1.2. Poplar

What makes poplar one of the most employed and proposed trees for use in green remediation processes in urban sites are its various and typical fundamental features, such as its huge root system, high resistance to and accumulation of pollutants, and its large biomass [62]. El Mahrouk et al. [63] reported on the uptake by black poplar (*Populus nigra* L.) of Cd, Cu, and Pb. The high BCF value revealed how the root system was the organ mainly involved in the accumulation of these pollutants in respect to the other plant organs. On the other hand, the authors recorded an increase in the TF values for Cd and Pb as their concentration increased in the soil.

Miletić et al. [64] showed that white poplar (*Populus alba* L.) accumulates some elements in concentrations considered toxic to plants, including Cr, Cu, Cd, Ni, and Zn. Moreover, a TF value > 1 for Zn, Cd, and Cu in regard to the phytoremediated sites highlighted the considerable phytoextractive capabilities of poplar. Finally, in regard to Canadian poplar leaves (*Populus × canadensis* Moench), Berezin & Olkova [65] observed an increase in Zn bioaccumulation by up to 2.3 times in respect to the minimum toxic level (100 mg kg^−1^) defined for this element [66].

#### 4.1.3. Willow

Willow (*Salix* sp.) is considered a good candidate for phytoremediation because it is a renewable energy tree and, subsequently, its biomass can be exploited for energy production [67]. The *Salix* genus also has positive traits like very easy propagation and management [68]. In a recent trial, Cao et al. [69] evaluated the growth and uptake capabilities of fifteen *Salix* clones in multi-contaminated (Cd, Zn, and Pb) soils, under non-flooded and flooded conditions. Except for Zn, which had higher accumulation in the leaves, all the other contaminants were mainly accumulated in the root system of all the studied clones.

Furthermore, Landberg et al. [70] published a study related to the remediation of an industrial contaminated site, using basket willow (*Salix viminalis* Balb.). Ten years after planting the willow trees, the authors reported a significant reduction in the concentrations for several diverse pollutants, both organics and inorganics.

#### 4.1.4. Sunflower

Sunflower (*Helianthus annuus* L.) is included in the category of hyperaccumulator species, due to its ability to store large amounts of different contaminants. For different Indian soil samples, collected from various industrial sites (e.g., plastic, paper, dye, etc.), Chauhan and Mathur [71] evaluated its HM uptake capability. The maximum accumulation values recorded were 158 mg kg^−1^ for Pb, 60 mg kg^−1^ for Cd, 167 mg kg^−1^ for Zn, and 102 mg kg^−1^ for Cu, etc. The authors also calculated the TF for each HM, which were all found to be <1, indicating a prevalent phytostabilization mechanism in regard to this hyperaccumulator.

#### 4.1.5. Other Plants Used for Phytoremediation

Several studies are mostly aimed at the remediation of inorganics; however, the mechanisms and processes involved in organics removal are still poorly investigated. Wyrwicka et al. [72] evaluated the organic remediation capacity of cucumber (*Cucumis sativus* L.) plants. In fact, it is known that the Cucurbitaceae family (e.g., cucumbers, pumpkins, and squashes) has shown a high level of potential in regard to the uptake and accumulation of organics. In this study, the authors highlighted the reduction in PCBs due to the use of cucumber plants, namely 38.47% for sewage sludge-treated soil and 27.62% for urban sediment-treated soil, respectively.

In regard to organic pollutants, Nissim et al. [73] reported on the capabilities of poplar, willow, eucalyptus, and sunflower to decrease the organic pollutant concentration in soil. For n-alkanes, willow (98.3%) and sunflower (97.3%) were highly effective at causing their reduction in sludge, while, among the other tested species, eucalyptus was the one that most reduced the concentration of PCBs (91.8%).

Another aspect that should be considered when choosing the best plant/tree species for phytoremediation is the use of native plants that are already living in a certain environment and, therefore, show those characteristics that favor their adaptation to the environment, thus reducing the risk of spreading alien plants in the environment. In fact, a better adaptation of the plants to different soils and environmental conditions affect the success of the remediation process, at least as much as all the other fundamental typical features of phytoremediation plants.

#### 4.1.6. Ornamental Plants

A fundamental aspect that must be considered in the context of phytoremediation is the aesthetic and landscape-related role that plants employed for the reclamation of brownfields must play to blend well into the urban setting in which they will be inserted.

In this regard, the possible use of ornamental plants, with environmental remediation capabilities, could represent an important frontier and ecological alternative, which is currently poorly investigated [74]. Watson and Bay [75] evaluated the phytoremediation capabilities and air pollution control of different plant species, planted on roadsides, initially for purely aesthetic purposes, in Trivandrum, the capital of the Kerala region (India), where air pollution exceeds the limits set by the National Ambient Air Quality (NAAQ) standards. Among the sixty-seven investigated plants, only six proved to be tolerant to the level of air pollution in the region; from the most tolerant to the least tolerant, we have American aloe (*Agave americana* L.), red cassia (*Cassia roxburghii* DC), cashew (*Anacardium occidentale* L.), golden shower (*Cassia fistula* L.), mango (*Mangifera indica* L.), and Ashoka tree [*Saraca asoca* (Roxb.) W.J.de Wilde].

Alternatively, de León et al. [76] explored the Al extraction and accumulation capacity of coleus [*Coleus scutellarioides* (L.) Benth], another ornamental plant. They showed that coleus was able to accumulate up to about 1450 mg kg^−1^ of this metal.

Among the direct advantages derived from the use of plants in urban phytoremediation, there is certainly the improvement of what is defined as ecosystem services.

Furthermore, the creation of ecological corridors using perennial plants results in a new habitat for different birds, insects, and other fauna species, simultaneously improving urban biodiversity.

**Table 2:** Potential bioaccumulation capabilities of the most common plant species used in phytoremediation.

Plants	Most Common Species	Features	Potential Bioaccumulation Capabilities	References
Poplar	*Populus nigra* L.; *Populus alba* L.; *Populus × canadensis* Moench; *Populus simonii* Carrière	Huge root system; high resistance and accumulation of HMs; large biomass; improves ecosystem services and mitigates pollutants	As (up to 2 mg kg^−1^) Cd (1–2 mg kg^−1^) Cr (4–7 mg kg^−1^) Cu (4–12 mg kg^−1^) Ni (3–9 mg kg^−1^) Pb (up to 407 mg kg^−1^) Zn (up to 223 mg kg^−1^)	[63,64,65,73,77]
Willow	*Salix × jiangsuensis*; *Salix wilsonii* Seemen; *Salix chaenomeloides* Kimura; *Salix cheilophila*; *Salix babylonica Linn. f. tortuosa Y.L.Chou*; *Salix viminalis* L.; *Salix alba* L.	Renewable energy crop; efficient, versatile, adaptable to environmental stress; rapid growth	Cd (up to 175 mg kg^−1^) Cr (up to 11 mg kg^−1^) Cu (up to 57 mg kg^−1^) Ni (up to 28 mg kg^−1^) Pb (up to 280 mg kg^−1^) Zn (up to 2977 mg kg^−1^)	[69,70,73,78]
Sunflower	*Helianthus annus* L.	Hyperaccumulator; rapid growth; high biomass	As (up to 3 mg kg^−1^) Cd (up to 60 mg kg^−1^) Cu (up to 102 mg kg^−1^) Pb (up to 158 mg kg^−1^) Zn (up to 214 mg kg^−1^)	[71,79,80]
Fescue	*Festuca arundinacea* Schreb.; *Festuca rubra* L.;	Rapid growth; wide diffusion, distribution, and variability on global scale; deep grass root penetration; dense and extensive fibrous root system	Cd (up to 2 mg kg^−1^) Cr (up to 11 mg kg^−1^) Cu (up to 28 mg kg^−1^) Ni (up to 8 mg kg^−1^) Pb (up to 55 mg kg^−1^) Zn (up to 562 mg kg^−1^)	[81,82,83,84]
Indian mustard	*Brassica juncea* L.	Extraction, sequestration and HM detoxification; high biomass; Hyperaccumulator	As (up to 0.5 mg Kg^−1^) Cd (over 0.3 mg kg^−1^) Cr (over 20 mg kg^−1^) Cu (over 40 mg kg^−1^) Ni (over 20 mg Kg^−1^) Pb (up to 40 mg Kg^−1^) Zn (up to 300 mg Kg^−1^)	[7,85,86,87,88,89]
Typha	*Typha latifolia* L.	Cosmopolitan plant, perennial and emergent macrophyte; wide diffusion; rapid growth; high biomass; adaptable to environmental stress	As (up to 1 mg Kg^−1^) Cd (up to 3 mg Kg^−1^) Cr (up to 25 mg Kg^−1^) Cu (48 ± 4 mg Kg^−1^) Ni (up to 38 mg Kg^−1^) Pb (up to 28 mg Kg^−1^) Zn (272 ± 18 mg Kg^−1^)	[90,91,92]

### 4.2. Microorganisms

Generally, when it comes to phytoremediation, the role of microorganisms is always perceived as secondary and underestimated to that of plants. Bacteria, fungi, and algae have a vital importance in this process. Suffice to say that microorganisms, due to their rapid growth and their possibility of being manipulated, are often used in association with plants to maximize the phytoremediation process [93]. Microorganisms can act in different ways during these processes. Some of them, called growth promoters, can produce phytohormones, siderophores, or solubilize nutrients that promote overall plant growth and health, resulting in an increase in its absorption capacity. Furthermore, through the excretion of chelators, they can facilitate the mobilization or sequestration of HMs, which are fundamental to phytoextraction processes. Moreover, thanks to the direct action of their enzymes, microorganisms can bio-transform or degrade contaminants, reducing their toxicity in soil. No less important, they also operate as a form of biocontrol for the plant, competing and inhibiting the activity of other pathogenic microorganisms.

Among the relevant bacterial strains, which are the most useful in regard to these processes, is certainly the *Bacillus* species. In particular, Rabani et al. [94] isolated and characterized, from Indian urban sites, two bacterial strains (*Bacillus licheniformis* and *Bacillus sonorensis*) capable of degrading naphthalene. Moreover, Liu et al. [95] reported an increase in the accumulation of Cd, Zn, Cu, and Pb by Alfred’s stonecrop (*Sedum alfredii* Hance) that was inoculated with *Bacillus subtilis* PY79. Similarly, Vaishnavi & Osborne [96] recorded an increase in the uptake of Cr, Pb, and Zn by vetiver (*Chrysopogon zizanioides* L.) that was inoculated with *Bacillus infantis* VITVJ8. In both cases, the presence of the microbial strains made the process more efficient than the presence of the plant alone. Also, the genus *Pseudomonas* has often been exploited in regard to bio-phytoremediation. Mukjang et al. [97] highlighted the role of *Pseudomonas aeruginosa* in crude oil degradation (up to over 80%). Similarly, Ummara et al. [98], using a bacterial consortium made up of *Pseudomonas aeruginosa*, *Burkholderia phytofirmans,* and *Acinetobacter junii,* inoculated in wheat (*Triticum aestivum* L.), reported a crude oil degradation rate of close to 50%.

In regard to plant–microorganism interactions, mycorrhizae come into play. They are fungi that form symbiotic associations with plant roots, increasing the surface area for absorbing water, nutrients, and, consequently, contaminants, but, above all, by improving the health of the plant’s root system; in fact, they facilitate colonization by strengthening the rhizospheric consortium [99].

Alinejad et al. [100] investigated how the symbiotic relationship between arbuscular mycorrhizae (*Funneliformis mosseae*) and rosemary roots (*Rosmarinus officinalis* Spenn.) increased Pb uptake (over 55%) in an urban site in Shiraz, a metropolis in south–central Iran. Moreover, Chane et al. [101] noted a significant increase in the degradation rates (48–60%) of PAHs caused by maize plants mycorrhized with a consortium of three white rot fungi (*Pleurotus ostreatus*, *Phanerochaete chrysosporium*, and *Irpex lacteus*) when compared to uninoculated plants, resulting in a degradation rate of 32% in 120 days. Other bacterial and fungal species, commonly used for the remediation of environmental matrices, belong to the genera *Rhizobium*, *Azospirillum*, *Arthrobacter*, *Enterobacter*, *Mycobacterium, Rhizophagus, Glomus*, and *Claroideoglomus,* etc., and other examples of microorganisms that play a role in phytoremediation are provided in the paragraph below.

## 5. Case Studies

Although phytoremediation has been widely studied (about 24,000 publications) and described in the last 20 years in scientific literature, demonstrating its effectiveness for different types of pollutants (inorganic, organic), few case studies (about 100 publications) and in situ applications in the urban context have been carried out worldwide. Therefore, our review examines several case studies that provide a general overview of phytoremediation applications, and it also illustrates its diverse and specific applications in urban environments, highlighting its adaptability and effectiveness in regard to different pollutants and its multifunctional benefits. Below, we have reported on a series of recent case studies (Table 3) and related results concerning different applications of phytoremediation techniques that we consider to be the most promising and innovative.

In a case study of Brazilian urban soils in a barite disposal area, the effectiveness of southern cattail (*Typha domingensis* Pers.) was demonstrated in regard to both phytostabilization and phytoaccumulation processes [102]. The authors optimized biomass production and Ba removal using a specific planting design (eight plants per square meter).

Another case study conducted in Wrocław, Poland, tackled air pollution mitigation using the “phytoremediation-by-design” method as a foundation for restructuring the area, integrating long-term phytoremediation practices into the site design process. This project involved the reorganization of an existing green space, already serving as a local playground and sports area [25]. The study employed phytoremediation as a method to enhance local air quality, focusing on the adsorption of particulate matter (PM_2.5_) and the modification of dispersion conditions. The design included the establishment of vegetative barriers, composed of trees and shrubs arranged in multiple rows, serving the dual purpose of reducing air pollution and acting as functional markers for the small-scale green area. The results confirmed that double-row shrub configurations significantly enhanced air purification, particularly under oblique wind conditions, showcasing how phytoremediation can be incorporated into urban planning for both environmental and recreational purposes.

In Vitoria-Gasteiz, Spain, the revitalization of abandoned peri-urban land using rapeseed (*Brassica napus* L.) and an organic amendment (BSM) derived from municipal solid waste was explored. This intervention not only improved plant growth and soil fertility, but also increased microbial activity and carbon sequestration, offering a low-cost and circular economy model for ecological remediation.

As already mentioned, a key role in phytoremediation is played by microorganisms that help plants in regard to the degradation of pollutants. In regard to this point, Mitter et al. [103] evaluated the effectiveness of four hydrocarbon-degrading endophytic bacterial strains, namely EA1-17 (*Stenotrophomonas* sp.), EA2-30 (*Flavobacterium* sp.), EA4-40 (*Pantoea* sp.), and EA6-5 (*Pseudomonas* sp.), which were previously isolated from an oil sands reclamation site. These strains were inoculated into white sweet clover (*Melilotus albus* Medik.) that was grown in soils amended with diesel at concentrations of 5.0, 10.0, and 20.0 g·kg^−1^. The results showed that bacterial inoculation alleviated diesel-induced phytotoxicity, as evidenced by significantly increased plant biomass, particularly at the 10.0 and 20.0 g·kg^−1^ diesel levels.

Traditional urban wastewater treatment plants have shown significant shortcomings regarding their poor ability to effectively eliminate both traditional and emergent pollutants. Treated wastewater, once released into the environment, can contain pathogenic microorganisms, posing a danger to public health. This risk is compounded by the presence and possible propagation of antibiotic-resistant bacteria (ARB) and their associated genes (ARGs). Through their study, Sousa et al. [104] developed a promising sustainable microalgae–bacteria (MBS) system for the bioremediation and disinfection of urban wastewater. This enabled the significant removal of NH_4_^+^, PO_4_^3−^, the coliform species present, and the antibiotic resistance genes, *sul1* and *bla*_TEM_.

Furthermore, Cheng et al. [105] compared the effectiveness of removing ARB and ARGs using a traditional wastewater treatment plant and a system employing extremophilic algae (*Galdieria sulphuraria*). The results showed how the algal system resulted in a reduction in erythromycin and sulfamethoxazole-resistant bacteria and even the elimination of several ARGs (*qnr*A, *qnr*B, *qnr*S, *sul*1, and *intI*1) in the effluent, performing much better than the conventional treatment plant.

Moreover, traditional wastewater treatment plants are unlikely to treat one of the most worrying classes of emerging pollutants in industrialized countries, per- and poly-fluoroalkyl substances (PFAS). Marchetto et al. [106] tested the *Synechocystis* removal capabilities in PFAS-enriched water. They revealed perfluorooctanoic acid (PFOA) and perfluorooctane sulfonate (PFOS) partial removal rates of 0.15 and 0.36 mg L^−1^ d^−1^, respectively. *Synechocystis* internalizes the PFOS into the cell, while the PFOA were transformed into sub-products, but this pathway is not yet known.

One of the limitations of constructed wetlands is the slowness of the process, which can take decades. In this sense, the integration of nanoparticles (e.g., zerovalent iron—ZVI nanoparticles, metal oxides, carbon, nanocomposites) offers a rapid and sustainable alternative with a low environmental impact, improving plant–microbe interactions, nutrient uptake, and pollutant degradation [107]. Majumdar et al. [108] evaluated the combined use of ZVI nanoparticles (at a dose of 20 mg kg^−1^) and four ornamental plants, namely vinca (*Catharanthus roseus* (L) G. Don), cosmos (*Cosmos bipinnatus* Cav.), globosa (*Gomphrena globose* L.), and balsamine (*Impatiens balsamina* L.), for the remediation of urban soil contaminated by As, Pb, and Hg. From this study, it emerged that treatment with cosmos and ZVI reduced the amount of As by 65.38%, Pb by 54.42%, and Hg by 61.67%, without any negative effects on plant growth. In conclusion, the selected case studies underscore the growing versatility and effectiveness of phytoremediation in urban contexts, ranging from soil and air purification to wastewater treatment, when integrated with innovative tools, such as planting design, microbial consortia, and nanotechnology. These approaches not only enhance contaminant removal, but also contribute to circular economy models, ecological restoration, and public health protection.

**Table 3:** Case studies carried out in urban areas worldwide.

Urban Area	Pollutant Type	Sampling Sites	References
Faisalabad City, Pakistan	Cu, Cd, Pb	Urban area with heavy traffic; residential area; building material manufacturing industry; weaving industry; pipe industry; cosmetic industrial area	[109,110]
Dhaka, Bangladesh	Cr, Pb, Cu, Zn	Riverbed sediments	[111]
Vian, Iran	Pb, Fe, Mn, Cu, Zn	Industrial area	[112]
Nitrastu, Spain	As, Cu, Zn	Urban brownfield	[113]
Trento, Italy	Landfill leachates (Cr, Cu, Zn, Al, Fe, Mn, Ni)	Municipal landfills	[114]
Baldegg, Switzerland		Catchment	[115]
Buiksloterham, Holland	Zn, Pb, Cu	Industrial area	[2]
Stockholm, Sweden	Cd, Cu, Pb, Zn	Stormwater ponds along a busy highway	[116]
Malmfjärden Bay, Sweden	As, Pb, Cu, Cr, Ni, Zn	Coastal area	[117]
Bor region, Serbia	High-molecular-weight polycyclic aromatic hydrocarbons (HMW PAHs)	Urban–industrial and residential areas	[118]
Warsaw, Poland	Cd, Cu, Cr, Ni, Pb, Zn	Urban area with heavy traffic	[119]
San Antonio and Baltimore, United States of America	Pb	Residential areas	[120]
San Diego, United States of America	Heavy metals and metalloids	Urban community garden	[121]
Canoas City, Brazil	Hydrocarbons and PAHs	Urban area	[122]

## 6. Advantages and Disadvantages of Phytoremediation in Urban Environments

### 6.1. Advantages of Phytoremediation

Phytoremediation is a promising solution due to its ecological robustness, cost effectiveness, and its ability to deal with a wide range of contaminants. Ecological solidity is achieved due to the fact that phytoremediation, unlike the totality of traditional remediation works, does not destroy the soil, but recovers it. In addition, it reduces the environmental impact and does not alter the habitats present on the site, positively affecting local biodiversity.

A great advantage of phytoremediation concerns both its minor costs and energy demands. However, the costs are closely linked to the specific context: it requires the collection of site-specific data, such as in relation to the characteristics of the landscape, the environmental conditions, and the local plant species present [123]. The same considerations were explained by Alshehri et al. [124] through their Life Cycle Assessment (LCA) of the phytoremediation technique as a nature-based solution in the urban context. It is noteworthy that the remediation costs are remedial objective and site specific; therefore, a direct comparison of the unit prices in the literature must be carried out.

Wan et al. [125] performed a cost–benefit analysis of a 2-year phytoremediation plan on contaminated soils (As, Cd, and Pb) in Huanjiang Maonan (China). As for the costs, USD 75,375.2/hm^2^ was spent on the construction of the work: 46.02% of this sum was part of the initial capital, while 53.98% was used for the operating costs. The initial capital included activities such as analysis of the pollution survey, the establishment of the remediation strategy, land preparation, nursery equipment, irrigation system, temporary warehouses, and equipment for the incineration of contaminated biomass at the end of the phytoremediation process. However, most of the initial capital was used to build the roads, bridges, and culverts needed to transport the materials and to build and operate the plant. The operational costs, on the other hand, included the cost of labor and materials, the use of large machines, and other direct costs (land rent, fuel and electricity costs) or indirect costs (staff salaries, administrative expenses, travel expenses). The benefit was estimated, however, considering the effects promoted by the phytoremediation process in terms of agricultural production and the improvement to human health and ecosystem services, which had been strongly altered by pollution. From this analysis, it emerged that the benefits induced by phytoremediation were able to compensate for the costs necessary to build the remediation plant in just seven years.

Similarly, Xu et al. [126], in China (Jinding town), considering four different technologies typically used for the remediation of contaminated sites (soil replacement, soil washing, solidification, and phytoremediation) found that, despite all the techniques being fruitful and advantageous, phytoremediation had the best cost–benefit ratio (benefit rate of 672.59% and a 96.90% reduction in the impact on health). However, one aspect that must be considered, although it is true that the construction of the phytoremediation plant is the cheapest of the four options, is the longer duration required by the process of remediation.

Hence, phytoremediation techniques stand out for their lower implementation costs and minimal ecological impact. However, they often require longer timeframes to achieve measurable results. In contrast, traditional physical–chemical methods offer, in some cases, faster and highly efficient pollutant removal, but at significantly higher financial and ecological costs. The presence of green spaces managed using phytoremediation can improve urban resilience to climate change, reducing the risk of urban flooding, thanks to the improvement of stormwater drainage and the landscape’s capacity to absorb air pollution [127]. Finally, the last aspect, which is not to be underestimated, is the reduced social and visual impact that results from phytoremediation that often contributes to the improvement of the urban or peri-urban landscape.

### 6.2. Disadvantages of Phytoremediation

Phytoremediation also has some disadvantages, the most significant of which is the large amount of land required for planting the vegetation species, especially in urban areas, where available space is often very limited [128]. Additionally, another limitation is related to the longer timeframes (as briefly discussed below), compared to other conventional treatment methods, needed for the plant species to interact with the contaminants, before their action can produce significant results. Finally, another aspect to consider is the need for the proper disposal of the contaminated biomass as a result of the remediation process, especially in the case of inorganic pollutants (e.g., HMs) that are not degradable. So, without adequate management, the reintroduction of these contaminants into the environment and trophic chain could occur, due to the natural decomposition of the biomass itself [129]. Nowadays, it is known that plants used for phytoextraction can be disposed of as hazardous waste in special landfills (compression landfills), or, where economically convenient, the practice of phytomining is implemented, with the recovery of precious and/or semi-precious metals from plants that have accumulated them in their organs [130]. Other methods of disposal and use can involve heat treatment that provides incineration, pyrolysis, and gasification, or through the use of the microbial treatment method, whereby microorganisms stabilize and humificate organic matter [131]. Moreover, once treated and free from contaminants, the contaminated plant biomass can be reused in the production of sustainable construction materials, such as biocomposite panels [132]. A further alternative in this context could be represented by the potential conversion of biomass into biofuels, such as gaseous fuels (biomethane and biohydrogen), deriving from thermochemical processes or anaerobic digestion, or liquid biofuels, using thermal and biochemical techniques [133]. To date, some projects have reported different strategies to counteract the problem of contaminated biomass management. Specifically, the Phy2Climate project implements the recovery of contaminated biomass to produce biofuel. As declared on their website (https://www.phy2climate.eu/the-project/ accessed on 25 June 2025), they use a Thermo-Catalytic Reforming (TCR) technology that combines an intermediate pyrolysis process with the subsequent catalytic reformation of the pyrolysis products. TCR technology has several fundamental advantages over conventional thermo-chemical processes for biofuel production from contaminated biomass, such as an already proven high feedstock flexibility. Furthermore, the Metalplant project (https://metalplant.com accessed on 25 June 2025) involves the use of plants for the extraction of nickel from soil and its recovery from the collected biomass through industrial processes. In conclusion, although the fate and recycling of plant biomass resulting from the phytoremediation process to date represent a well-established problem and a disadvantage in terms of the implementation of the process itself, a sustainable approach, with proper planning and management of the problem, could lead, in the near future, to the affirmation of this green remediation technology over other traditional and more detrimental approaches.

## 7. Final Consideration

This review discussed the application of phytoremediation in urban areas, demonstrating its effectiveness, cost efficiency, and its applicability in various urban contexts and scenarios, as discussed above, relative to the different case studies. In fact, the choice of the most appropriate phytoremediation strategies and plant species is critical. For instance, in an urban park that is the subject of reclamation, the use of phytostabilization would be advisable. This approach prevents the translocation of contaminants to the aerial parts of the plants, thereby safeguarding human and animal health. Moreover, phytoremediation offers significant ecological and social benefits. Plant species commonly used in phytoremediation, such as willows (*Salix* spp.) and poplars (*Populus* spp.), contribute to improving urban air quality through the air deposition and filtration of particulate matter, or the absorption of pollutants. Moreover, these trees provide shade, helping to mitigate the urban heat island effect, and also creating a cooler microclimate in cities. The resulting green spaces not only enhance the environmental conditions, but also promote the well-being of residents, offering areas for recreation and relaxation, which contribute to their mental and physical health. Phytoremediation can also support biodiversity, by using perennial and native plant species. These trees create habitats for insects, birds, and other wildlife, enhancing urban biodiversity and promoting several ecosystem services. The restored land becomes sustainable areas that support a variety of life forms, further increasing their environmental value.

Furthermore, the integration of phytoremediation into urban planning contributes to the resilience of cities in the face of climate change by improving stormwater management, reducing air pollution, and enhancing carbon sequestration. Additionally, the transformation and the consequent redevelopment of contaminated sites into attractive green areas can significantly increase property value, making such interventions economically advantageous for local communities and investors. However, this review also highlights the limitations that need to be addressed to improve the efficacy of phytoremediation. One of the main challenges is the long amount of time required for plants to interact with contaminants and achieve significant remediation results. This often makes phytoremediation unsuitable for high-priority sites requiring rapid clean up. Finally, the space requirements for planting, particularly in densely populated urban areas, often restrict the feasibility of large-scale applications. To overcome these limitations, future research should focus on accelerating the remediation process using genetically engineered plants or plant–microbe partnerships. Developing hybrid approaches that combine phytoremediation with either advanced chemical or physical methods or both could enhance its efficiency.

Another aspect to consider is that stakeholder approval is essential for phytoremediation to be recognized as a credible alternative to conventional remediation.

Addressing this critical concern requires an integrated approach capable of balancing economic sustainability, environmental protection, and the quality-of-life of communities.

In conclusion, while phytoremediation still presents some challenges, its ecological, social, and economic benefits make it a highly valuable tool for urban environmental restoration and, addressing its limitations through research, innovation, and policy support will pave the way for its broader implementation, ensuring cleaner, healthier, and more resilient urban environments.

## Data Availability

All the data are available from the corresponding author upon reasonable request.
